# Possibilities for Using Pluripotent Stem Cells for Restoring Damaged Eye Retinal Pigment Epithelium

**Published:** 2018

**Authors:** A. E. Kharitonov, A. V. Surdina, O. S. Lebedeva, A. N. Bogomazova, M. A. Lagarkova

**Affiliations:** Federal Research and Clinical Center of Physical-Chemical Medicine of Federal Medical Biological Agency, Malaya Pirogovskaya Str., 1a, Moscow, 119435, Russia

**Keywords:** Retinal pigment epithelium, differentiation, embryonic stem cells, induced pluripotent stem cells, cell therapy, clinical trials

## Abstract

The retinal pigment epithelium is a monolayer of pigmented, hexagonal cells connected by tight junctions. These cells compose part of the outer blood-retina barrier, protect the eye from excessive light, have important secretory functions, and support the function of photoreceptors, ensuring the coordination of a variety of regulatory mechanisms. It is the degeneration of the pigment epithelium that is the root cause of many retinal degenerative diseases. The search for reliable cell sources for the transplantation of retinal pigment epithelium is of extreme urgency. Pluripotent stem cells (embryonic stem or induced pluripotent) can be differentiated with high efficiency into the pigment epithelium of the retina, which opens up possibilities for cellular therapy in macular degeneration and can slow down the development of pathology and, perhaps, restore a patient's vision. Pioneering clinical trials on transplantation of retinal pigment epithelial cells differentiated from pluripotent stem cells in the United States and Japan confirmed the need for developing and optimizing such approaches to cell therapy. For effective use, pigment epithelial cells differentiated from pluripotent stem cells should have a set of functional properties characteristic of such cells *in vivo. *This review summarizes the current state of preclinical and clinical studies in the field of retinal pigment epithelial transplantation therapy. We also discuss different differentiation protocols based on data in the literature and our own data, and the problems holding back the widespread therapeutic application of retinal pigment epithelium differentiated from pluripotent stem cells.

## INTRODUCTION

**Fig. 1:**
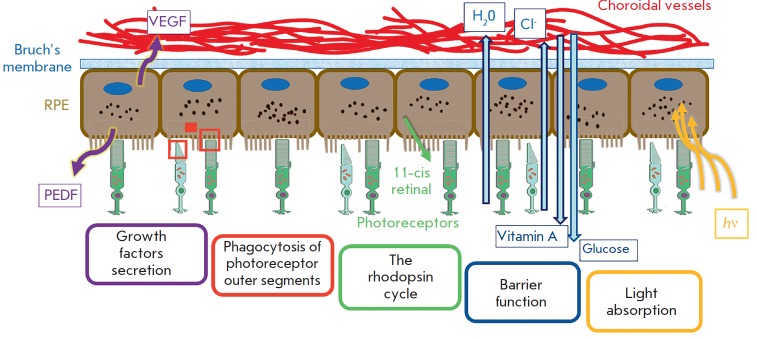
Functions of retinal pigment epithelium (according to [4] with changes)

Retinal pigment epithelium (RPE) is formed by a monolayer of hexagonal epithelial cells with a large number of melanosomes containing a pigment's melanin (*Fig. 1*). The inner layer of the five-layer Bruch’s membrane serves as the basal membrane for pigment epithelium. The nuclei of RPE cells are located closer to the basal pole, which contains fewer melanosomes. The RPE apical pole contains many melanosomes and microvilli (cilia), which “envelop” the outer segments of the photoreceptor cells. There are long and short microvilli. Short microvilli are connected to the ends of the outer segments of the photoreceptors, whereas the long ones are located between the outer segments [1]. Each RPE cell is in contact with 20–55 photoreceptors [2] in the area of the macula. The space between the RPE microvilli and the outer segments of the photoreceptors is filled with matrix, which, together with the microvilli, ensures close fitting of the retina to the RPE.

Functions of the RPE:

Absorption of light. The RPE melanosomes absorb most of the light uncaptured by photoreceptors. It prevents reflection and diffusion of light across the retina, which allows for maintaining contrast and clarity of an image. Under the influence of light, melanosomes migrate to the apical side of cells, into the surroundings of the outer light-sensing segments of the photoreceptors microvilli. In the dark, melanosomes return back to the central part of the cell with the assistance of microfilaments and a hormone known as melanotropin. The light-absorbing function is provided mainly by long microvilli [3]. In addition, the RPE helps dissipate heat in the retina, which is released as a result of lightcapture and the process of visual phototransduction [2].

Phagocytosis. The RPE cells carry out phagocytosis of used photoreceptor discs [5]. Each RPE cell daily phagocytizes 2–4 thousand used disks [6].

Implementation of the visual cycle. The disks of the outer segments of the photoreceptors contain large opsine proteins and are responsible for the absorption of light. It is synthesized in the inner segments and transported to the outer segments. Rhodopsin is necessary for the visual cycle and consists of opsin bound to 11-*cis*-retinal. When light has been captured, retinal isomerizes from 11-*cis*-retinal into *trans*-retinal and then converts to *trans*-retinol. During the visual cycle, photoreceptors find themselves unable to convert *trans*-retinol back to 11-*cis*-retinal; therefore, it is transported to the RPE for re-isomerization and subsequently returns to the photoreceptors [1].

Barrier function. Providing a selective supply of the necessary nutrients to the photoreceptors of the vascular membrane and removal of degradation products in the opposite direction. The RPE is the second part of the hematoretinal barrier, which prevents large molecules from entering the retina from the choriocapillaries. The first part of this barrier is the endothelium of retinal capillaries [3, 5, 6].

Secretion of hormones and growth factors. Polarized RPE cells secrete various cytokines and growth factors in different directions, which is very important for the functioning of choriocapillaries and retina. For example, secreted from the basal side of the RPE cells VEGF is vital for choriocapillaries, whereas PEDF and TGF-β, which are secreted mainly by the apical side of the RPE cells, are required in the subretinal space [1, 2].

There is no doubt that the RPE plays an important role in sustaining photoreceptors and that proper functioning of the photoreceptors is impossible without a healthy RPE.

## DISEASES RELATED TO RPE DEGENERATION

The most common diseases involved in RPE degeneration are age-related macular degeneration (AMD) of the retina and pigment retinitis (PR) [6, 7]. These two diseases are the main causes of blindness in Western countries. To date, there are no satisfactory ways to treat them, since the retina and RPE do not regenerate, and only in the case of a “wet” form of AMD can the course of the disease be slowed down through anti-VEGF therapy [8].

AMD is a multifactorial disease that can develop under the influence of genetic factors, aging, and lifestyle (smoking, body mass index, diet) [1]. AMD has “dry” and “wet” forms. In the dry form of AMD, small amorphous deposits containing fats and proteins, known as druses, accumulate under the macula between the inner layer of the Bruch’s membrane and the basal membrane of RPE [9]. This leads to local inflammation caused by oxidative stress [1]. Over time, the communication between RPE and photoreceptors is lost, which leads to the deterioration of central vision. This form of AMD is the most common one and occurs in approximately 90% of people with this disease. In the case of the wet form of AMD, new blood vessels appear in parts of the macula where they should not be present. This causes the destruction of the macula structure and leads to a rapid loss of central vision. Although this type of AMD occurs in about 10% of people with this disease, it accounts for 90% of the AMD-related decline in vision.

Pigmented retinitis (PR) is the main cause of blindness among children and adolescents. The PR prevalence is 1 case per 3,500 people [8, 10]. This hereditary disease often leads to blindness and is characterized by progressing dysfunction and death of the rods. In some cases, PR is accompanied by damage to RPE. It was found that about 50 mutated genes are associated with PR. Among them, there are genes encoding proteins associated with the transmission of a light signal, retinoid cycle, cell adhesion, and cytoskeleton [10]. The most common type of PR is caused by mutations in the gene encoding opsin [11]. The early stages of PR involve degeneration of the rods, which results in patients losing night and peripheral vision. At the late stages of PR, patients develop the tunnel syndrome and cones also begin to die, leading to serious issues [1].

The Stargardt’s and Best’s diseases, forms of heritable juvenile macular degeneration, are less common than PR and AMD. Stargardt’s macular dystrophy is caused by mutations in the *ABCA4 *gene, which lead to the accumulation of di-retinoid-pyridinium ethanolamine (A2E) and its modification in RPE cells. These toxins demolish the pigment epithelium and follow the death of the photoreceptors of the retina’s macula, accompanied by a loss of central vision [12, 13]. At the moment, there are no ways to stop the loss of vision caused by Stargardt’s macular degeneration [14]. There is a whole series of so-called “Stargardt’s-like diseases,” caused by mutations in the genes *CNGB3*, *ELOVL4*, and *PROM1 *[14, 15].

Best’s disease is an autosomal-dominant hereditary disease caused by mutations in the *BEST1 *gene that encodes transmembrane protein bestrophin-1. This protein is part of the basolateral plasma membrane of RPE, but its function has not been fully elucidated. It is known that Best’s disease alters the transport of chloride ions and disrupts fluid transport through RPE and the accumulation of metabolites (for example, lipofuscin) and fluid between the Bruch’s membrane and the RPE/photoreceptor complex. These processes cause the death of photoreceptors and loss of central vision [1, 12]. Best’s disease was the first among retinal diseases whose cellular model was created with the help of patient-specific IPSCs. The model was used to demonstrate, at the molecular level, that processing in mutant RPE of the outer segments of photoreceptors is disrupted and that the rhodopsin transformation cycle is slowed down [16].

## POTENTIAL OF CELLULAR THERAPY IN DISEASES OF THE EYE

There are various strategies for using autologous and allogeneic material for transplantation in patients with AMD [6] and other diseases associated with degenerative processes in the retina. Three main strategies are used for clinical trials with autologous material: translocation of the macula (e.g., [17]), autotransplantation of the RPE-choroid flap (e.g., [18]), and subretinal injection of a suspension of autologous RPE cells (e.g., [19]).

Meurs et al. described the experience of autotransplantation of peripheral RPE to seven patients with AMD. Postoperative follow-up lasting a year revealed no significant improvement in visual acuity, although three patients positively assessed their condition. Two patients reported a decline in vision, which, according to the authors, could have been associated with postoperative retinal detachment and proliferative vitreoretinopathy [20].

Falkner-Radler et al. compared the efficiency of autotransplantation of the RPE-choroid flap and subretinal injection of a RPE suspension in two groups of seven patients, each. Based on the results of a 24-month follow-up of the patients from both groups, no statistically significant differences were observed in the best corrected visual acuity test (BCVA). However, the individual results in the groups were ambiguous. For example, both improvement of visual function and deterioration of visual acuity were reported by individual patients in both the first and second groups [21].

In 2012, van Zeeburg et al. reported on the ability of a transplanted RPE-choroid flap to sustain the function of the macula over a long period of follow-up with a relatively low level of complications and relapses. The work was based on a 7-year postoperative follow-up period of 130 patients who underwent autologous transplantation of the RPE-choroid flap (a total of 133 eyes) in the period from 2001 to 2006 [22]. The study and its main findings were criticized in a review by Seiler and Aramant (2012), who pointed out the absence of a control group and the low number of eyes that were actually followed up after the surgery (only 9 eyes at year 7 of the follow-up) [23].

Therefore, the issue of the effectiveness and stability of results in an autotransplantation of RPE remains controversial. The undoubted advantage of the method is the lack of histocompatibility issues and absence of a need for immunosuppressive therapy. On the other hand, autotransplantation may lead to unpredictable results [24]. It cannot be excluded that cells transplanted from a site unaffected by the degeneration already had hidden morphofunctional changes. In addition, the difficulty of obtaining material for autotransplantation from healthy areas limits the possibilities of treatment at late stages of the disease.

Donor tissue can be another source of cells for RPE transplantation in patients with macular degeneration, as long as there is donor-recipient histocompatibility. As a rule, fetal material is transplanted, although there are reports of transplantation of allogeneic tissue from adult donors.

Allogeneic fetal material was first used for RPE transplantation in 1999 in the treatment of 16 patients [25]. At the time, immunosuppressive therapy was not used in the postoperative period and 75% of the patients experienced slow rejection of the transplanted tissue. The first report on the transplantation of RPE from an adult donor was published in 2001 [26]. That surgery was performed on an 85-year-old patient who died 4 months after the procedure. The surgery itself did not lead to an improvement in visual indices. Therefore, the first clinical transplantations of RPE from allogenic donor tissues proved rather unsuccessful.

The first encouraging results of allogeneic transplantation were obtained by American surgeon Norman D. Radtke. In 2004, he published a report on the transplantation of the fetal neuroretina/RPE complex to a 64-year-old woman with pigmentary degeneration of the retina (PDR) [27]. The surgery led to an improvement in visual acuity in the patient during a 5-year follow-up period. Later, in 2008, the same surgery was performed by the same surgeon in 10 patients with PDG, and in seven of them it improved visual acuity. In one patient, visual acuity did not change in the postoperative period, while in two patients vision deteriorated [28].

In 2007, another group of ophthalmic surgeons performed transplantation of allogeneic RPE tissue from adults to 12 patients with exudative macular degeneration. The patients received a course of immunosuppressive therapy lasting up to 6 months. The postoperative follow-up during the first year showed improvement in visual acuity, reading, and other parameters of visual function, although statistical methods did not confirm the observed differences [24].

Thus, despite early promise, outcomes for the transplantation of autologous or donor tissue in AMD have been controversial. In the case of autotransplantation, there is a risk associated with a surgical intervention involving the gathering of healthy tissue in a degeneration-free area of the retina and further manipulations involving the introduction of an autograft into the macula area [29]. In addition, there is the possibility of continuing degradation of the autotransplanted healthy tissue. Allogeneic transplantation is inevitably accompanied by problems related to obtaining donor material, donor-recipient histocompatibility, and the need for immunosuppressive therapy, which in turn is associated with a variety of side effects.

Let us mention that descriptions of experiments with mesenchymal stem cell (MSC) transplantation for the treatment of eye diseases are beyond the scope of this review. We would only mention that it is already generally accepted that, in this case, MSCs can only have a paracrine effect, since these cells do not possess the ability to differentiate beyond the mesodermal germinal layer [30, 31].

At the end of the 20th century, cultures of mouse and human embryonic stem cells (ESC) were obtained from the internal cell mass of blastocysts [32, 33]. In 2006, induced pluripotent stem cells (IPSC), ESC analogues, were created by genetic reprogramming of differentiated cells [34]]. ESC and IPSC are pluripotent; i.e., they are capable of unlimited growth and self-renewal, as well as differentiation into cells of any type. Pluripotent stem cells (PSC) can provide a solution to the problem of finding a source of cells for transplantation, which researchers have encountered in clinical trials of autologous and allogeneic RPE transplantation. In recent years, several protocols for directional differentiation of ESC and IPSC into RPE have been developed and some of them have been tested in clinical trials [5, 35-39]. It should be noted that the anatomical and morphological features of the eye (relatively small size, organ pairing, well-developed methods of diagnosis and instrumental monitoring, possible immune privilege and presence of the hematoretinal barrier) make it a convenient target for refining the technology of delivery of material in the case of cell therapy involving pluripotent cells derivatives [40].

## DIRECTED DIFFERENTIATION OF PSCS INTO RPE

Directed differentiation of IPSCs into RPE and further use of the obtained material in clinical practice are of interest to many researchers (e.g., [11, 41]). We compare different differentiation protocols, their effectiveness, and time costs. Let’s briefly consider some of the protocols that currently seem most effective to us.

Insignificant amounts of retinal pigment epithelial cells can be formed during spontaneous differentiation of human pluripotent stem cells [35]. If FGF2, which is necessary to maintain the pluripotent state in culture, is removed from the culture medium, human pluripotent stem cells cultured on a mouse embryonic fibroblast substrate, matrigel, polylysine, or laminin become capable of forming pigment epithelial cells [35, 42, 43]. After 10–12 weeks of spontaneous differentiation, small pigmented regions form, which are then mechanically separated from the rest of the cell mass, yielding an almost pure cell culture of pigment epithelium ( > 99% purity). Various modifications of culture medium composition and time of differentiation make it possible to increase the yield of pigment epithelial cells [44, 45]. However, effective directional differentiation protocols are required to obtain cultures that are enriched in retinal pigment epithelial cells without a labor-intensive mechanical selection of pigmented colonies. Recent studies have shown that it is possible to produce cells of retinal pigment epithelium from IPSCs and ESCs *in vitro *which are morphologically and functionally similar to such cells *in vivo*. For example, Leach et al. compared the effectiveness of spontaneous and directed differentiation protocols into RPE using five different IPSC lines obtained from different donors and different cell types. It has been shown that the source of donor cells, the method of reprogramming, and the protocol used can affect the possibility of effective differentiation [46], which once again underscores the need for a standardization of the procedure for obtaining RPE from PSCs for clinical use.

One of the first directional differentiations of PSC into RPE was performed by Hirami et al. [47]. Mouse and human IPSCs in a suspension culture were treated with Wnt and Nodal antagonists to promoted differentiation into pigment epithelium.

Since RPE cells differentiate from the neuroectoderm and share common characteristics with neuronal retina cells *in vivo*, a two-stage differentiation protocol was developed to produce pigment epithelial cells from neuroepithelial precursors [48-51]. The ESCs aggregates were initially cultured in a suspension in a medium for neuroepithelial differentiation. Then, neuroepithelial progenitors were expanded and differentiated into putative pigment epithelial cells by replacing FGF2 in the culture medium with B27 additive. The first cells, similar to retinal pigment epithelial cells, appeared after 4 weeks of differentiation, and after 8 weeks the number of cells suitable for subcultivation became significant. This two-step method is more effective than the method of spontaneous differentiation.

Based on the role of nicotinamide (NIC) in metabolism, survival, plasticity and cell differentiation, Idelson et al. investigated the effect of NIC on the differentiation of ESCs into pigment epithelial cells [5]. To induce a differentiation into RPE cells, ESC clusters obtained with collagenase were cultured in a suspension in a ESC medium supplemented with a serum substitute, NIC, and with or without activin A (a member of the TGF-β superfamily that directs the differentiation of the eyeball in embryogenesis) [52]. Pigmented areas appeared 4 weeks after the induction, and about half of the clusters were pigmented when cultured in media containing both NIC and activin A. It has been shown that NIC in the presence of activin A effectively induces and increases the efficiency of differentiation of ESCs into pigment epithelial cells.

The protocols described above assume a lengthy time of differentiation and produce a low-purity population, which requires additional laborious manipulations to purify the cells of the desired type. Buchholz et al. proposed a faster and more effective protocol. This method of directed differentiation of ESC into pigment epithelial cells is based on a combination of factors inducing retinal differentiation (IGF1, Noggin, Dkk1, bFGF), and other factors (NIC, activin A, SU5402 and vasoactive intestinal peptide (VIP)), and all factors are added at different, strictly defined time points [44]. Already 14 days after the initiation of the differentiation, about 80% of the cells in the culture were pigment epithelial cells. According to the authors, this protocol can be used to rapidly build up the large quantities of cells necessary for transplantation due to its high efficiency and speed (there are minor variations of this protocol proposed by other authors, e.g. Geng et al. [53]). A similar protocol was proposed by Foltz and Clegg, who used CHIR99021 instead of VIP [54].

To identify new compounds that contribute to the differentiation of IPSC into RPE, a quantitative PCR screening of RPE differentiation markers in a IPSC culture was performed by analyzing a chemical library [39]. As a result, chetomin, a substance that potentially activates differentiation, was identified. Then, using a reporter construct (GFP under the control of a RPE-specific tyrosinase enhancer), it was confirmed that chetomin, an inhibitor of the hypoxia-induced factor (HIF), significantly increased the differentiation of PSCs into RPE. The combination of chetomin with nicotinamide led to the differentiation of more than 50% of IPSCs into RPE. The molecular pathways by which chetomin promotes the differentiation into RPE are still unknown.

To obtain retinal cells, Zhu et al. also used inducers such as IWR1, SB431542, and IGF1, and they obtained functional photoreceptors and retinal pigment epithelium from IPSCs in compliance with GMP standards. It has been shown that the obtained derivatives can integrate the retina of immunodeficient mice [55].

**Fig. 2:**
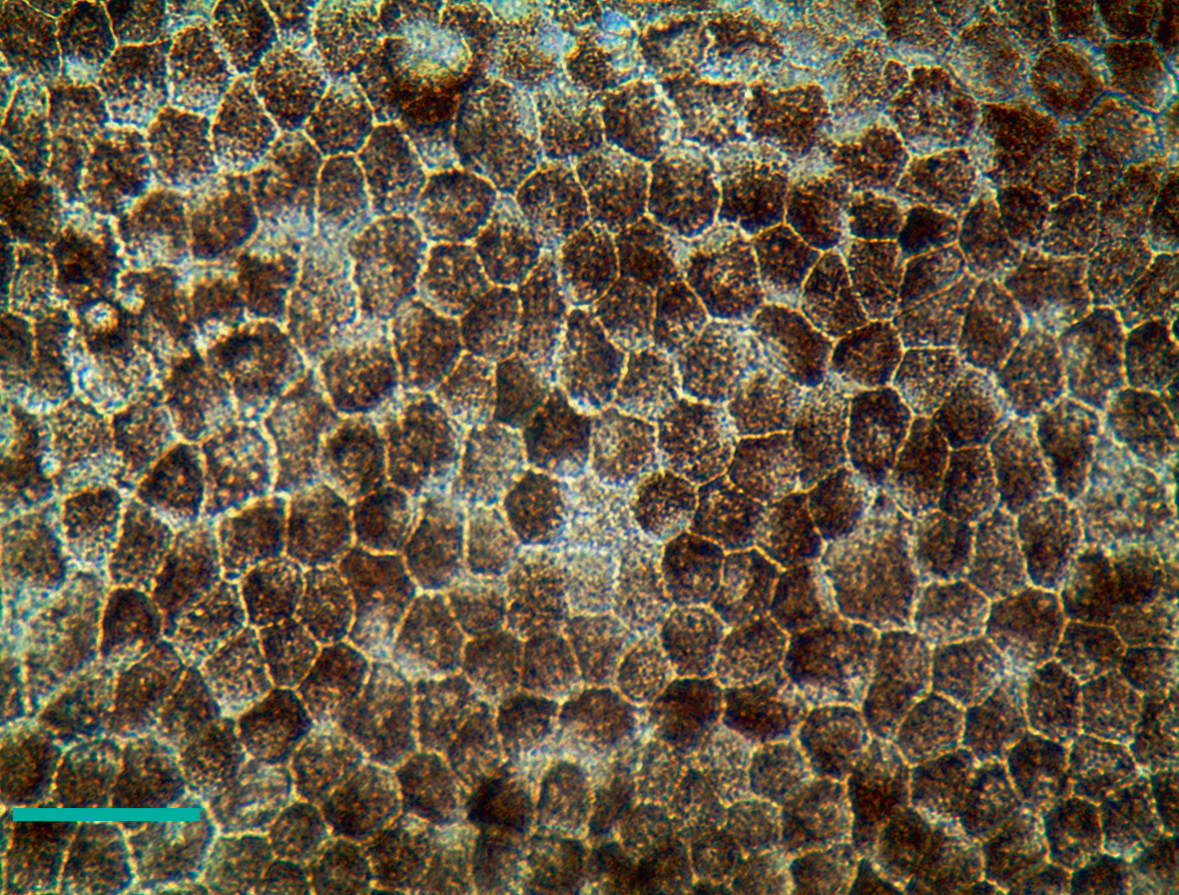
RPE cells differentiated from the IPSC of a healthy donor. The cells were cultured for 3 months in a Transwell chamber. Phase contrast. The scale bar is 100 mM

In our laboratory, several methods of differentiating PSCs into RPE have been tested. A comparison of several differentiation protocols led us to the following conclusions:

1. In our experience, the protocol [39] with chetomin and nicotinamide is the best protocol, working reliably for all tested IPSC and ESC lines. In this case, the addition of activin to the medium is undesirable, since it reduces the survival rate of cells and the effectiveness of the directed differentiation (unpublished data). Differentiation takes at least 30 days, but this “loss of time” is compensated for by a large number of pigmented cells obtained and their subsequent rapid proliferation.

2. Pigment epithelium cells are extremely sensitive to the extracellular matrix; their survival, maturation rate, and completeness of phenotypic and functional characteristics typical of this type of cells *in vivo *depend on the type and quality of the matrix [56, 57]. The natural substrate for the cells of retinal pigment epithelium is Bruch’s membrane. According to our experimental data, matrigel is the most suitable substrate for a rapid growth of immature, rapidly dividing RPE cells in laboratory. Most likely, in order to achieve full hexagonal morphology and correct polarization of RPE, it is necessary for the liquid to wash the layer of the epithelial cells from both sides, apical and basal. In order to achieve that, the RPE cells are usually cultivated in chambers, transwells, where the medium is located above and under the membrane on which the cells grow. A representative photograph of RPE differentiated from IPSCs and having a characteristic morphology and pigmentation is shown in *Fig. 2*.

One of the most important functional characteristics of retinal pigment epithelial cells is the ability to secrete PEDF and VEGF, and also to form an extracellular matrix [4], interact with the outer segments of the photoreceptor, and phagocytize them [38]. Therefore, these physiological properties are usually tested to prove the functionality of the differentiated RPE. The expression of the genes that encode typical RPE proteins (e.g., RPE 65, BEST1, tyrosinase, MITF1, ZO- 1, etc.) is also checked. Another very important characteristic of RPE is its transepithelial potential, which reflects the barrier properties of the epithelium. This potential can be measured with a conductometer.

The functionality of differentiated RPE *in vivo *is confirmed in animal models, primarily in rats of the RCS (Royal College of Surgeons) line with recessively inherited dystrophy of the retina [5, 38, 58] and in albino rabbits [59]. Numerous studies have shown that in animals after transplantation of the pigment epithelium, its histological and physiological features are preserved. Electroretinography was used to demonstrate the functionality of the transplanted RPE (review [60]).

## CLINICAL TRIALS OF RPE DIFFERENTIATED FROM PSCS

The first clinical trials using RPE cells derived from pluripotent stem cells were performed by American specialists Schwartz et al. in 2011. ESC of a MA09 line were used to obtain RPE. This trial was registered in the ClinicalTrials.gov database under the IDs NCT01345006 (Stargardt’s disease) and NCT01344993 (atrophic AMD). At the first stage, one patient with Stargardt’s disease and one patient with atrophic AMD received subretinal injections of 50,000 RPE cells. The results of the postoperative follow-up revealed no side effects over the course of 4 months, including hyperproliferation and oncogeneity. Visual acuity improved in both patients based on objective indicators [61]. At the next stage, a clinical cohort of 18 patients was given different doses of the transplantation material: 50 ×10^3^, 100 ×10^3^ and 150×10^3^ cells. During a period of postoperative follow-up of 22 months, an increase in retinal pigmentation was noted in 13 patients; improvement of vision was noted in 10 patients [14].

The protocol by Schwartz et al. was used in 2012 by Korean ophthalmologists in the NCT01625559 clinical trial. Minor modifications of the protocol concerned screening for oncogenicity and a scheme of postoperative immunosuppressive therapy. Two patients with Stargardt’s disease and two patients with AMD received a subretinal injection of 40 × 10^3^ RPE cells differentiated from the ESC of a MA09 line. Based on the Early Treatment Diabetic Retinopathy Study and a BCVA test, visual improvement was registered in three patients. In one patient, immunosuppressive therapy was discontinued 4 weeks after the surgery due to the development of side effects, and the state of the retina returned to its preoperative level. In general, the feasibility and preliminary safety of cell therapy with RPE differentiated from ESC in macular degeneration of various etiologies have been confirmed. However, it has been noted that further observations, clinical trials, and studies are required [12].

In 2012, Pfizer launched a phase I clinical trial of a transplantation of ESC-derived RPE grown on a polyester membrane (NCT01691261) at University College London. In that trial, the transplantation was performed in patients with the wet form of AMD with progressive loss of vision. Currently, patients who participated in the phase I are being recruited to the next clinical trial (NCT03102138), which will involve a 4-year follow-up and safety assessment of the earlier conducted transplantation.

In 2015, three different universities in China announced the start of phase I clinical trials of subretinal transplantation of RPE differentiated from ESCs (NCT02749734, NCT02755428, NCT03046407). In each trial, the surgery will be performed on 10–15 patients with various forms of retinal dystrophy. The studies will assess safety and the clinical effect of the transplantation.

Since 2015, the Federal University of São Paulo (Brazil) under the leadership of Professor Rubens Belfort has been conducting a two-stage clinical trial which examines the feasibility of transplantation of ESC-derived RPE (NCT02903576). The first stage will include transplantation of PRE in the form of a suspension; and the second stage, in the form of a monolayer on a polymer substrate. The purpose of the trial is to compare the efficacy of the two methods of transplantation, as well as to assess its safety and applicability in clinical practice.

Currently, Regenerative Patch Technologies, led by Jane Lebkowski, is recruiting patients in the United States to participate in the Phase I/II clinical trial of transplantation of ESC-derived RPE on a parylene membrane. The trial will include 20 patients, distributed into two groups based on the stage of “dry” AMD (NCT02590692).

A trial of the commercial cell productOpRegen®, a suspension of RPE cells derived from human ESCs, has been started by U.S. and Israeli medical teams. In this trial, 15 patients with atrophic AMD will undergo transplantation of the product into the subretinal space, followed by vitrectomy (NCT02286089).

An analysis of the ClinicalTrials.gov database shows that the main objects of clinical trials across the world are cells obtained from ESCs. The first – and so far only – published clinical trial of RPE differentiated from IPSCs has been carried out in Japan [62]. The bias in favor of ESCs can be attributed to greater reservations on the part of the biomedical community regarding IPSCs. The production of IPSCs requires a much higher number of manipulations per cell than the production of a ESC line. There are doubts regarding the stability of the IPSC genome, in the completeness of reprogramming and differentiation. IPSCs are also not quite as widely represented in clinical trials, since it is a relatively new type of cells; they were first obtained in 2006, whereas mouse and human ESC cells have been studied 25 and 8 years longer, respectively. In our opinion, one could expect an increase in the number of trials of IPSC products within the next three to four years, especially in Japan and China.

According to Federal Law of June 23, 2016, No. 180- FL, human ESCs and fetal cells are not allowed to be used as a source of cellular products. Regardless of the opinion of the authors about this prohibition, Russian researchers are faced with the fact that IPSCs remain essentially the only source of cells for producing RPE.

The first clinical trial of RPE differentiated from IPSCs was conducted in Japan [62]. The Japanese doctors transplanted a monolayer of RPE differentiated from IPSCs to a 70-year-old patient with neovascular age-related AMD. The patient underwent surgery which included the removal of the neovascular membrane and transplantation of the autologous RPE under the retina. A year after the surgery, the transplanted layer of RPE remained intact, visual acuity did not improve, but it did not worsen either, and cystoid macular edema was present. Autologous IPSCs were obtained using nonintegrating plasmid vectors and differentiated into RPE according to a previously published protocol that allows obtaining functional RPE [62]. The quality and safety of the IPSCs and the RPE cells obtained from them were carefully analyzed before the transplantation. In addition to the assessment of the morphology and expression of the relevant markers, the authors performed karyotyping with traditional GTG-banding and a full-genomic SNP-analysis, as well as full-genomic sequencing, and full-genome analysis of transcriptome and DNA methylation. The absence of RPE tumorigenicity was demonstrated by transplanting the RPE to immunodeficient NOG mice.

Pioneering transplantation of RPE differentiated from IPSCs certainly became a huge step in regenerative medicine. However, it also left many unresolved issues. It should be noted that, initially, the PRE transplantation should have been performed in two patients, but for one of them IPSCs did not pass quality control due to identified CNV that appeared during the reprogramming. In addition, 10 out of the 20 IPSC clones selected for further analysis contained plasmids integrated into the genome: i.e., the preparation of IPSCs using plasmids should not be recognized as the safest way of reprogramming [62]. Other means of production could be nonintegrable viruses, *in vitro *synthesized RNA, and reprogramming with small molecules [63-65].

So far, the international community has not developed unambiguous recommendations either on methods for obtaining or on the necessary and sufficient methods of characterization of cells derived from PSCs. A prerequisite for the full-scale application of PSC-differentiated derivatives is the assessment of the effect of various protocols of their production and subsequent cultivation on the genetic and epigenetic stability of cells with sequencing and the methylation profiling of the whole genome, analyses of the expression, as well as elucidation of the molecular basis, of possible aberrations [66]. In addition, surgical transplantation and instruments are now being actively developed, which should make this procedure as safe as possible for patients [67].

Autologous transplantation of IPSC derivatives is a very expensive and long-term method. As mentioned above, allogeneic transplantation requires immunosuppression. It has seemed that the solution to this problem could be the development of IPSC banks from healthy donors with homozygous genes of the main histocompatibility complex HLA [68]. Each such homozygote will be compatible with any heterozygote in which there is one allele of the same haplotype. It is estimated that the 20 most common homozygous HLA haplotypes of the European population, identified after screening of 26,000 individuals, will be suitable for 50% of the population [68]. The creation of such a bank of IPSC lines began as a national initiative in Japan in 2012, and IPSCs with the most common “Japanese” haplotypes are already available for use at the IPSC Research and Application Center in Kyoto [69]. However, the work published last year [70] slightly dampened optimism regarding this approach. It turned out that when immune cells heterozygous for HLA interact with HLA-homozygous graft cells, the recipient’s NK cells are able to cause the rejection of cells derived from homozygous IPSC by recognizing the “absence of one’s own” [70]. This issue requires further study.

## CONCLUSION

So far, there have been no proven methods for restoring or improving vision in patients with retinal degeneration. One such method can be the transplantation of retinal tissues, in particular pigment epithelium. The approach associated with the transplantation of RPE derived from human PSCs has already been used in several clinical trials. Retinal pigment epithelium could be obtained by directional differentiation of human ESCs and human IPSCs and selected based on morphological criteria and the accumulation of brown pigment granules. However, wide application of PRE differentiated from PSCs requireds addressing many different issues. In particular, methods for sorting the RPE, necessary and sufficient procedures for proving the equivalence of the differentiated cells to the RPE cells, methods and protocols of cell delivery, surgery technologies and criteria for selecting patients for RPE transplantation should be elaborated. For example, progression of the disease is associated with degeneration of both RPE and photoreceptors, and, therefore, it becomes necessary to transplant both RPE and the photoreceptors in order to achieve an effective clinical outcome. In addition, personalized therapy with autologous cells is unlikely to become a generally available medical procedure in the coming decades due to its labor-intensity and the high cost associated with obtaining and differentiating patient-specific IPSCs. The search for approaches to allogeneic transplantation of IPSC-derivatives would make it possible to reduce the cost and accelerate the production of RPE cells for transplantation in the degeneration of the retina.

## Abbreviations

AMD: age-related macular degeneration
IPSCs: induced pluripotent stem cells
BCVA: best-corrected visual acuity
MSCs: mesenchymal stem cells
PDR: pigmentary degeneration of the retina
PSCs: pluripotent stem cells
RPE: retinal pigment epithelium
ESCs: embryonic stem cells
ABCA4: ATP-binding cassette sub-family A (ABC1) member 4
BEST-1: Bestrophin 1
CNV: copy number variations
NIC: nicotinamide
NK: natural killers
PEDF: pigment epithelium-derived factor
bFGF: basic fibroblast growth factor
VEGF: vascular endothelial growth factor
